# Supplementary material for: Chemoattraction of Neoplastic Glial Cells with CXCL10, CCL2 and CCL11 as a Paradigm for a Promising Therapeutic Approach for Primary Brain Tumors
Source: Int J Mol Sci. 2021 Nov 10;22(22):12150. doi: 10.3390/ijms222212150 (PMC8626037; doi:10.3390/ijms222212150)
Supplement: Supplementary file 1 [file ijms-22-12150-s001.zip › ijms-1424483-supplementary.pdf]

Supplementary Materials for

Chemoattraction of neoplastic glial cells with CXCL10, CCL2 and CCL11 as a paradigm for a promising therapeutic approach for primary brain tumors

Laurence Déry[1,*], Gabriel Charest[2], Brigitte Guérin[1], Mohsen Akbari[3,4], David Fortin[2]

*Corresponding author. Email: Laurence.Dery3@USherbrooke.ca

This PDF file includes:

Figures. S1 to S4
Table S1

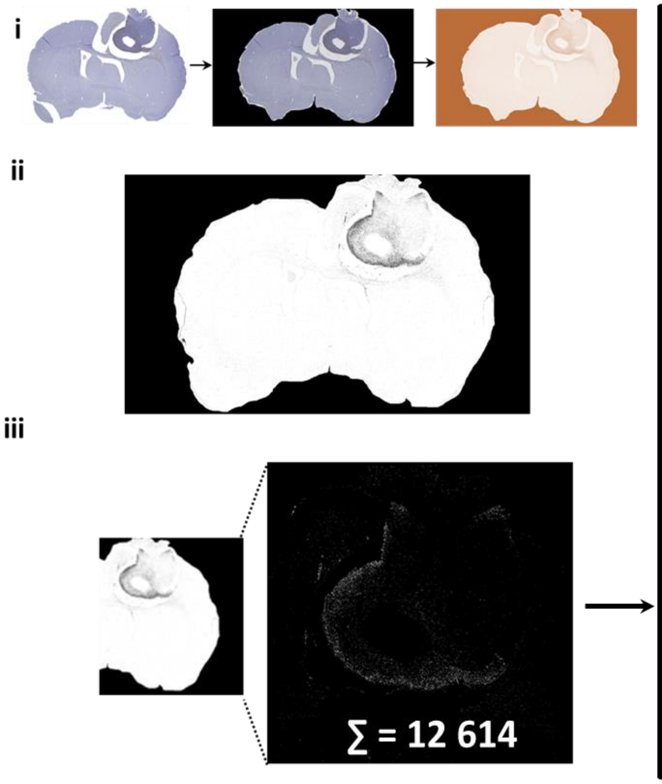

		Left hemisphere	Right hemisphere
GFAP	Ctl intra	17 638	24 942
	Ctl contro	27 931	38 347
	CXCL10 intra	57 113	64 983
	CXCL10 contro	95 468	82 949
	CCL2 intra	21 380	37 084
	CCL2 contro	67 230	70 737
	CCL11 intra	61 259	81 246
	CCL11 contro	47 195	76 922
	3 c.a. intra	39 906	73 871
	3 c.a. contro	53 767	54 521
Nestin	Ctl intra	201	6 283
	Ctl contro	1 554	13 158
	CXCL10 intra	466	12 614
	CXCL10 contro	3 347	14 594
	CCL2 intra	511	9 676
	CCL2 contro	1 983	18 631
	CCL11 intra	1 340	8 581
	CCL11 contro	2 631	34 171
	3 c.a. intra	456	8 448
	3 c.a. contro	2 808	10 339
Ki-67	Ctl intra	3	94
	Ctl contro	8	54
	CXCL10 intra	33	1 011
	CXCL10 contro	98	310
	CCL2 intra	14	305
	CCL2 contro	164	625
	CCL11 intra	39	418
	CCL11 contro	44	810
	3 c.a. intra	17	290
	3 c.a. contro	89	981

Figure S1.

IHC image quantification procedure in Image J/ Fiji software. (i) The outline of the immunohistochemical image was freehand selected and deleted, making it black and an image deconvolution was performed in H&E/ DAB; (ii) Threshold was applied according to the marker, for GFAP: value < 125 = 0; if not = 1, Nestin: value < 145 = 0; if not = 1 and ki67: value < 175 = 0; if not =1; (iii) the resulting image was split to separate each hemisphere. Then, each image was inverted to obtain IHC staining in white pixel. Finally, the sum of the points of each image has been carried out.

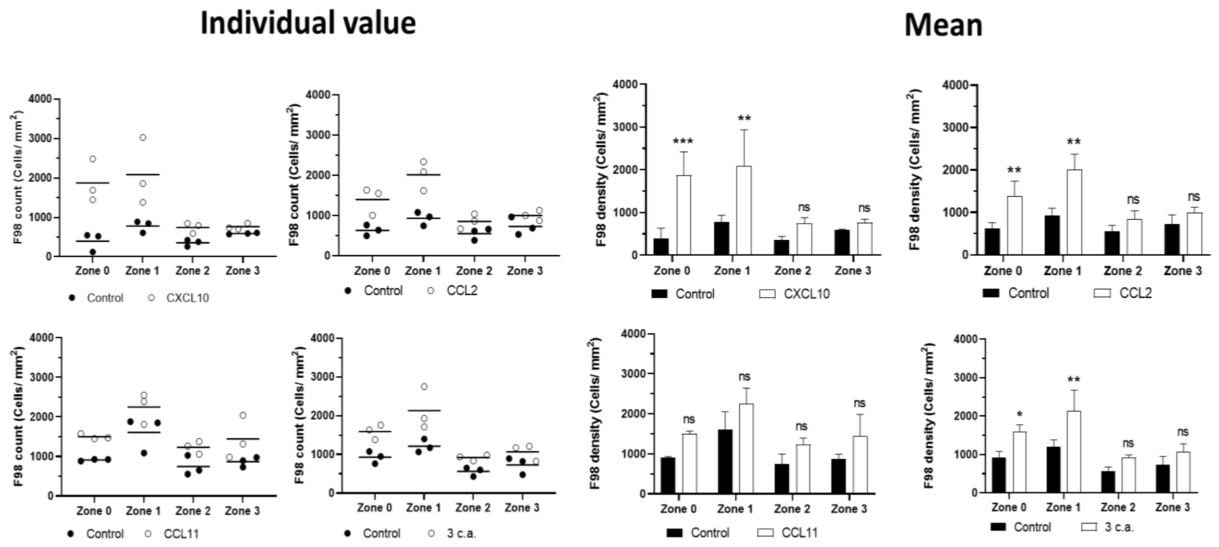

Figure S2A.

Individual and mean values for chemoattraction potential of each ligand with F98 tumor cells.
*, $p < 0.05$, **, $p < 0.01$, ***, $p < 0.001$, ns = not significant.

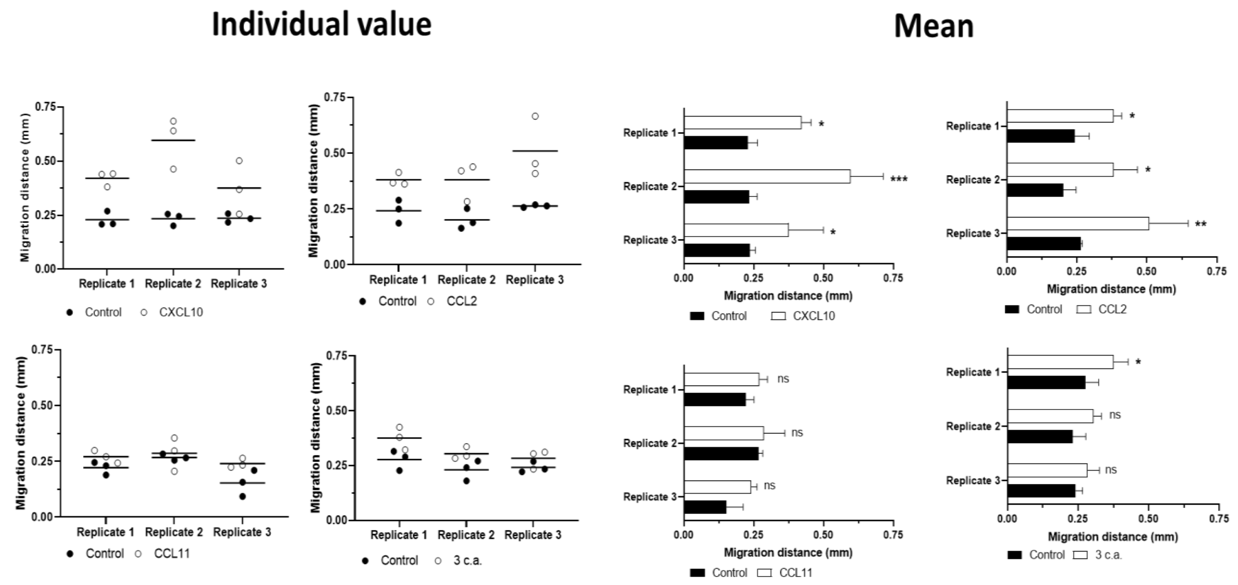

Figure S2B.

Individual and mean values for migration distance inside the drop (zone 0) of each ligand for F98 tumor cells. *, $p < 0.05$, **, $p < 0.01$, ***, $p < 0.001$, ns = not significant.

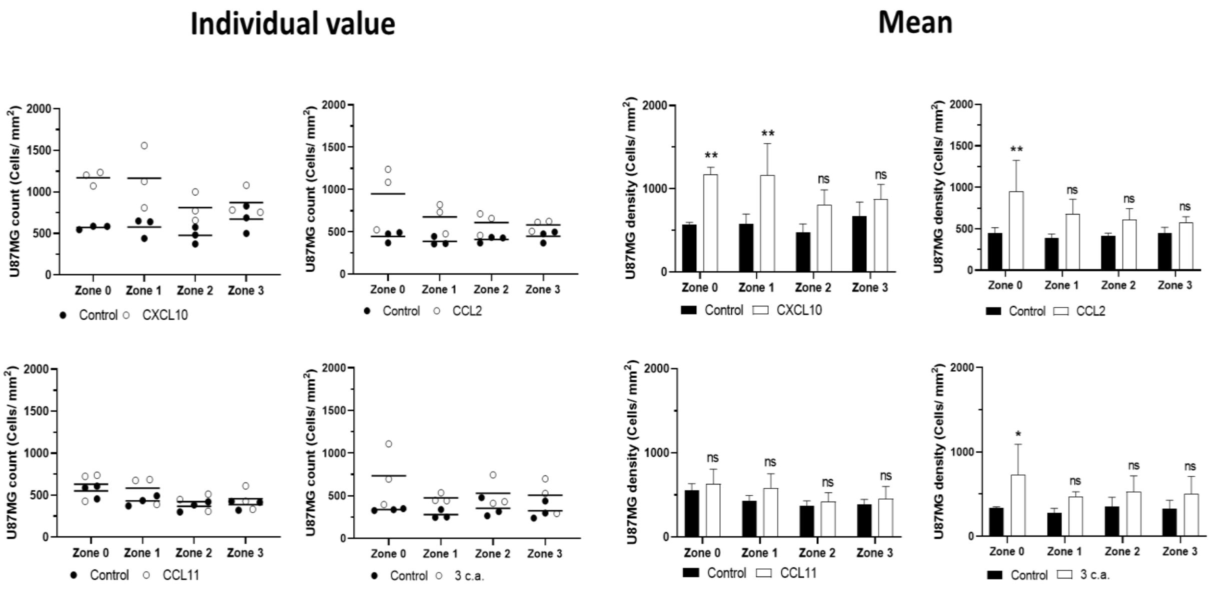

Figure S3A.

Individual and mean values for chemoattraction potential of each ligand with U87MG tumor cells.

*, $p < 0.05$, **, $p < 0.01$, ns = not significant.

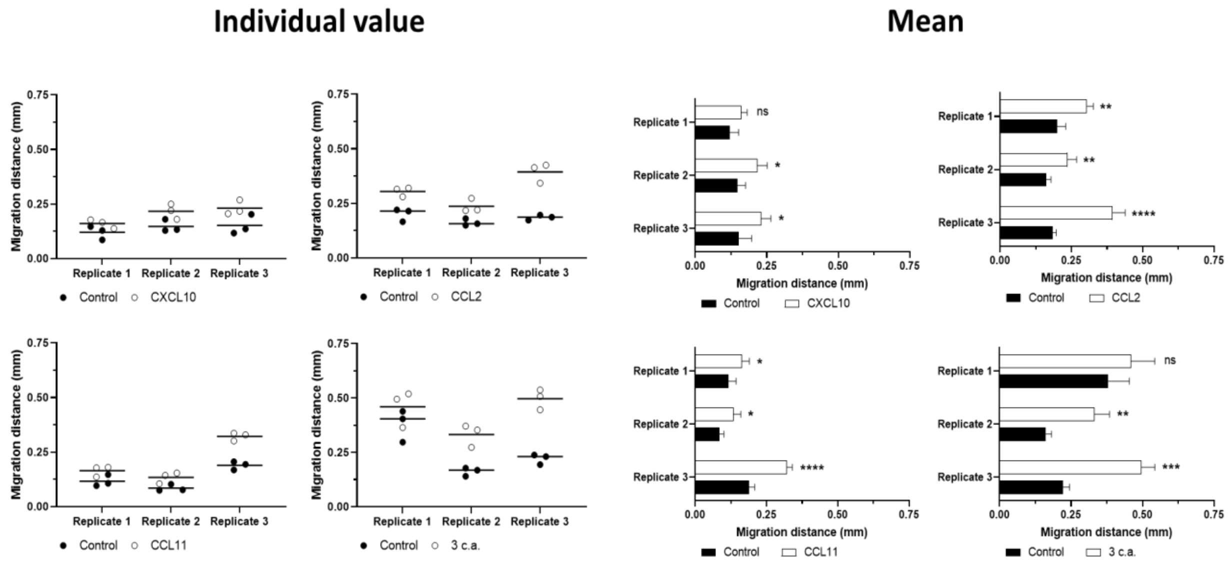

Figure S3B.

Individual and mean values for migration distance inside the drop (zone 0) of each ligand for U87MG tumor cells. *, $p < 0.05$, **, $p < 0.01$, ***, $p < 0.001$, ****, $p < 0.0001$, ns = not significant.

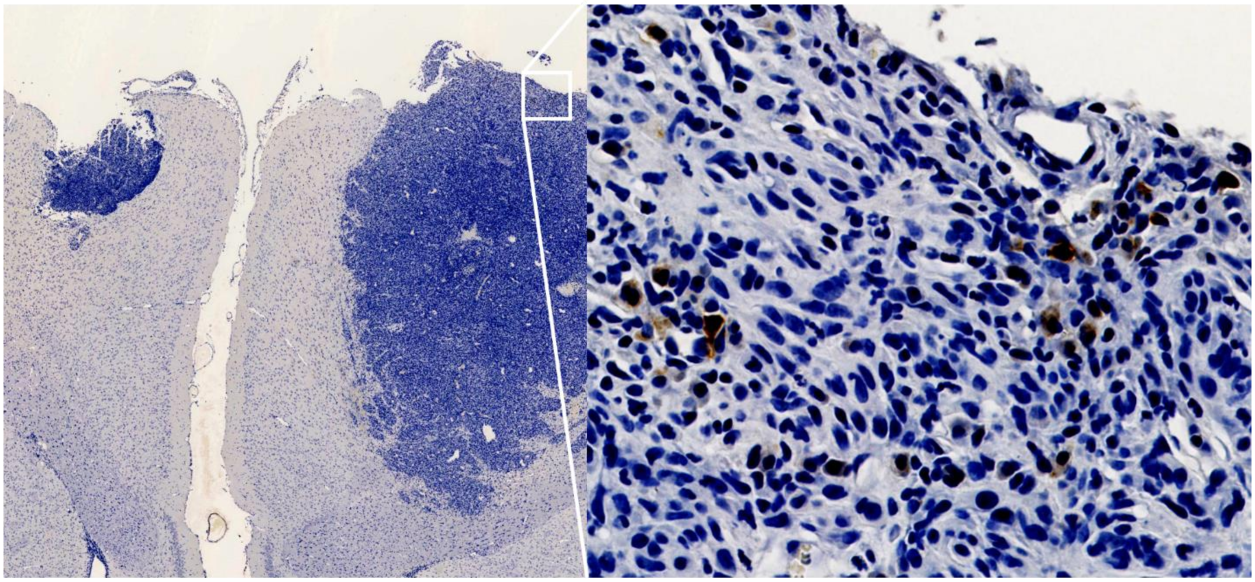

Figure S4.

Immunohistochemical labeling of CD68 on controlateral implantation of GlioGel containing a combination of the three chemoattractants. Unusual intracellular cell marking, mostly at the superficial periphery of the tumor implantation site.

	Controlateral			Intratumoral		
Control<br>**44** ± 7.483<br>(n = 8)	*	65 ± 19.07 (n = 7)	CXCL10<br>***	7 ± 2.340 (n = 7)	****	Control<br>**37** ± 5.855<br>(n = 8)
	****	84 ± 14.24 (n = 8)	CCL2<br>****	28 ± 8.064 (n = 9)	*	
	*	61 ± 16.06 (n = 8)	CCL11<br>***	25 ± 4.086 (n = 8)	***	
	ns	53 ± 16.01 (n = 7)	Combination<br>(3 c.a.)<br>**	24 ± 10.15 (n = 7)	**	

Table S1.

Mean values of peritumoral cluster with respective standard deviation and significance level for each data set. *, $p < 0.05$, **, $p < 0.01$, ***, $p < 0.001$, ****, $p < 0.0001$, ns = not significant.
